# Beyond reductionism: the emerging holistic paradigm in indirect control of pathogen infection

**DOI:** 10.3389/fmicb.2025.1638634

**Published:** 2025-09-18

**Authors:** Seonghan Jang, Dajeong Kim, Hwi Won Seo, Choong-Min Ryu

**Affiliations:** ^1^Infectious Disease Research Center, KRIBB, Daejeon, Republic of Korea; ^2^Department of Pediatrics, School of Medicine, University of California San Diego, La Jolla, CA, United States

**Keywords:** indirect pathogen control, helper microbe, beneficiary microbe, reductionism, holism, PGPR

## Abstract

Microbial research has often emphasized direct interactions between pathogens and other microbes, leading to the discovery of antibiotics and biological control agents. However, such approaches frequently overlook the complexity of microbial ecosystems and show limited effectiveness in real-world settings. Indirect interactions, where non-pathogenic microbes influence pathogen behavior through ecological networks, offer an alternative strategy for controlling infectious diseases. We hypothesize that targeting microbes support or influence pathogen activity, rather than the pathogen itself, can lead to more effective and sustainable disease control. Indirect modulation of pathogen behavior through ecological networks may reduce virulence, persistence, and resistance development. Recent studies in both animal and plant systems support this idea, showing that manipulating non-pathogenic microbial relationships can suppress disease occurrence more efficiently than direct intervention. Focusing on indirect ecological relationships allows for a more comprehensive understanding of pathogen dynamics and presents new opportunities for sustainable disease management.

## Introduction

Nature ensures that every organism, from viruses to humans, exists within a web of relationships that form the bedrock for survival and evolution. These interactions have been categorized into eight types: keystone prediction, exploitation competition, apparent competition, indirect mutualism, indirect commensalism, habit facilitation, trophic cascade, indirect defense, and apparent prediction (Moon et al., 2010). In fields such as entomology, ecology, and human behavior, such interactions are pivotal research topics; however, due to their inherent complexity, most studies have focused on one-to-one interactions, leaving indirect interactions relatively underexplored. Consequently, the concept of “interaction” has often been oversimplified to signify “direct interaction”, neglecting the intricate dynamics of “indirect interactions”.

In microbiology, while the significance of indirect microbe-microbe interactions is acknowledged, their practical applications and implications of these complex dynamics remain largely underdeveloped. Traditionally, research has concentrated on direct interactions; examples include the discovery of antibiotics through inhibition of pathogenic microbes *in vitro*. This approach, involving Petri dishes and test tubes to screen for microbial inhibition, has been the standard method for identifying potential antibiotics that control diseases in humans, animals, and plants (Trepka et al., 2024). However, direct inhibition of target microbes by antibiotics has contributed to the rise of antimicrobial resistance and persistence (Windels et al., 2024). In plant pathology, microbe-based biological control agents against microbial pathogens have achieved limited success due to inconsistencies in understanding the multi-layered interactions from laboratory screening to field application (Niu et al., 2020). This focus on direct interaction represents a reductionist perspective, where antagonistic microbes are typically selected based on straightforward, one-to-one interactions, rather than the intricate, multi-faceted interactions that occur in natural environments.

The holobiont concept, viewing hosts and their associated microbiomes as a unified ecological entity, highlights the necessity of considering the intricate networks in surrounding pathogens (Simon et al., 2019). However, the specific mechanisms and applications of indirect ecological interactions in pathogen control are not yet fully understood. The grouping of these interactions under the term “indirect interactions” aims to provide a framework for exploring how non-pathogenic microbes can influence pathogen behavior and infection indirectly, through multi-trophic and multi-species networks.

The development of next-generation sequencing technologies has revolutionized microbiome research, allowing for high-throughput and culture-independent analysis of microbial communities (Human Microbiome Project, 2012). These advances have enabled researchers to explore not only the taxonomic composition but also the functional potential and ecological dynamics of microbial populations. Amplicon sequencing of the 16S rRNA gene, shotgun metagenomics, and metatranscriptomics have become standard approaches to profile microbial diversity and infer interactions across a range of environments, including the soil, rhizosphere, gut, and skin.

To move beyond simple co-occurrence patterns, computational tools such as CoNet, SparCC, and SPIEC-EASI have been developed to reconstruct microbial association networks (Rottjers, 2018). These methods leverage statistical and probabilistic models to predict interactions based on abundance correlations while accounting for the compositional nature of microbiome data. Some tools, such as MENA or FlashWeave, have incorporated ecological filtering or time-series data to strengthen inference of potential interactions. Through these methods, researchers can identify central nodes or “hub” taxa that disproportionately influence community structure and function.

Despite these advances, many studies have focused primarily on keystone or dominant microbes, often neglecting low-abundance but functionally significant “helper” microbes. These microbes may not be central in correlation networks but can exert critical ecological functions, such as nutrient provisioning or stress mitigation, that indirectly influence community dynamics. Recognizing and characterizing these helper microbes is essential for a deeper understanding of multi-layered microbial interactions and for designing more effective microbial interventions in both medical and agricultural systems.

However, despite growing recognition of their ecological significance, indirect microbial interactions remain underutilized in practical pathogen control strategies. Most existing studies continue to emphasize direct antagonism, overlooking how microbe–microbe networks contribute to pathogen persistence, virulence, or suppression. This presents a critical gap in the current literature, particularly regarding the functional classification and application of microbes that indirectly influence pathogenic outcomes. To address this, the present study introduces a conceptual framework for understanding indirect pathogen inhibition, synthesizes recent examples across host systems, and proposes a multi-layered model to categorize levels of indirect microbial influence.

## Microbial helper theory

Endosymbiotic microbes that confer advantages on plants or animals undergo significant genomic reduction through evolution, thereby adapting to their specific host environments. Similarly, certain free-living microbes, known as beneficiaries, possess substantially reduced genomes, making them incapable of surviving or proliferating independently. These beneficiaries rely on essential nutrients produced by neighboring microbes, termed helpers, for survival.

Prominent examples of a beneficiary-helper interaction include mycorrhizal fungi and a green alga. Mycorrhizal fungi intimately interact with rhizosphere-dwelling bacterial symbionts known as mycorrhiza helper bacteria (MHB), which facilitate symbiosis between fungi and plants (Shi et al., 2023). MHB can directly affect fungal physiology or indirectly support symbiosis by suppressing plant defense mechanisms. A recent study revealed that a helper bacterium, *Mycetocola*, protects microalgae against an antagonistic *Pseudomonas* bacterium by detoxifying harmful compounds (Hermenau et al., 2020).

Recent studies highlight interactions between human pathogens and their helper bacteria, revealing their role in enhancing pathogen virulence. In the gut, *Enterococcus faecalis* increases the pathogenicity of enterohaemorrhagic *Escherichia coli* by upregulating the type 3 secretion system through cross-feeding adenine (Martins et al., 2024). Similarly, *Bacteroides* spp. promotes pathogen expansion in colitis by providing monomeric nutrients (Culp, 2023). In the skin microbiome, the commensal *Cutibacterium acnes* promotes biofilm formation by *Staphylococcus aureus* through coproporphyrin III-induced aggregation (Byrd et al., 2018). Beyond commensals, other pathogens can also facilitate the virulence of other pathogens. For example, *Pseudomonas aeruginosa* exacerbates *S. aureus*-induced skin lesions by upregulating toxin expression (Hotterbeekx et al., 2017). Given that these pathogen-helper bacteria can enhance pathogen pathogenicity directly, they should also be considered potential targets for treating infectious diseases. During these bacteria-bacteria interactions, helpers typically support growth of other bacteria by supplying essential nutrients or mitigating environmental and biological stresses (Morris, 2015).

Although these examples illustrate direct interactions between helpers and pathogens, they also suggest the intricate web of indirect interactions within microbial communities. *In situ* conditions, such as those in the rhizosphere and human intestine, present scenarios involving multiple microbes that interact through secondary or tertiary networks. These interactions can influence pathogen indirectly, affecting factors like virulence, persistence, and susceptibility to treatment. Understanding these indirect interactions is crucial for developing holistic strategies for pathogen control.

Nonetheless, investigating indirect interactions poses substantial challenges. Methodological biases in computational analyses can overemphasize certain species, and environmental variables can affect microbial dynamics. Furthermore, the complexity of the multi-species networks makes it difficult to predict outcomes reliably. Despite these obstacles, exploring indirect interactions offers the potential to uncover novel targets for intervention and to develop more sustainable approaches to disease management (Li et al., 2022; Na et al., 2024).

## Indirect inhibition of animal and plant pathogens on skin and others

**Figure 1:**
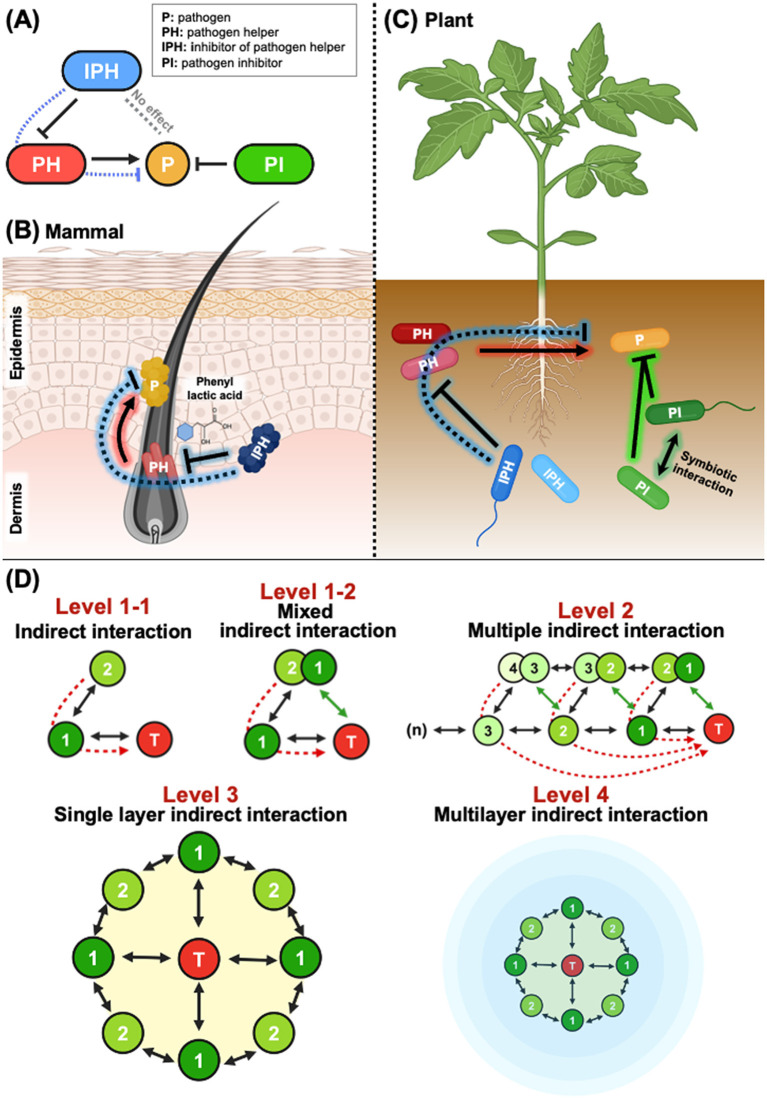
Indirect microbial interactions. **(A)** Dynamics of direct and indirect microbial interactions. In both animals and plants, microbial communities comprise four key bacterial types: pathogens (P), pathogen helpers (HP), inhibitors of pathogen helpers (IPH), and pathogen inhibitors (PI). PI bacteria can suppress pathogens directly, while PH bacteria enhance the growth or virulence of pathogens. IPH bacteria, by suppressing PH bacteria, inhibit pathogens indirectly, without affecting them directly. **(B)** Indirect control of *S. aureus* via suppression of PH bacteria. In mammalian skin, the pathogen *S. aureus* is controlled indirectly by targeting the PH bacterium *C. acnes*. The IPH bacterium produces phenyl lactic acid, which suppresses *C. acnes*, thereby reducing *S. aureus-*induced skin inflammation. **(C)** Indirect control of phytopathogen *R. solanacearum* in tomato plants. Similar to animal systems, phytopathogens are influenced by PH bacteria. *R. solanacearum* can be suppressed directly by PI bacteria through synergistic effects. In addition, IPH bacteria in the rhizosphere suppress PH bacteria, thereby controlling *R. solanacearum* indirectly. Overall, both in animal and plant systems, indirect control of pathogens proves more effective than direct control. **(D)** Overview of direct and indirect microbial interactions. At Level 1-1, Bacterium 1 interacts directly with the target microbe (T) (black line), while Bacterium 2 influences the target indirectly by interacting with Bacterium 1 (dashed red line). The distinction between Level 1-1 and Level 1-2 lies in whether Bacterium 2 establishes a direct relationship with the target microbe. In Level 1-2, if Bacterium 2 also interacts directly with T (green line), it transitions from being an indirect interactor to a direct interactor, essentially becoming another Bacterium 1. At Level 2, the complexity increases as multiple bacteria interact in a sequence, where the indirect effects on the target microbe accumulate as these interactions overlap. In Level 3, a single layer of bacteria surrounds the target microbe. Here, each bacterium impacts the target both directly and indirectly through interactions with other bacteria in the same layer. This single-layer network of interactions shows how multiple bacteria can collectively influence the target microbe. Finally, at Level 4, complex multilayer interactions are depicted, representing a more natural or *in situ* state in which bacteria are interconnected through multiple layers. These multilayer structures form a comprehensive network that collectively influences the target microbe, illustrating how microbial interactions can evolve from simple direct interactions to complex, multi-layered networks in more realistic environments.

The human skin microbiome is a complex and dynamic ecosystem composed of both commensal and potentially pathogenic microorganisms (Grice, 2011). Skin-associated microbes interact with each other and with host immune responses, playing a crucial role in maintaining skin homeostasis or contributing to disease development under dysbiotic conditions. A recent study demonstrated that targeting helper bacteria can effectively attenuate skin infectious disease (Na et al., 2024). The study identified three distinct classified bacterial types [pathogen (P), pathogen helper (PH), and inhibitor of pathogen helper (IPH)] within facemask-contaminated microbiomes (Figure 1A). Co-infection with PH bacteria (non-virulent commensal *C. acnes*) facilitated the growth and pathogenicity of *S. aureus* (P), thereby worsening skin inflammation. However, inhibiting PH with phenyl lactic acid produced by IPH improved *S. aureus*-induced inflammation indirectly, whereas direct pathogen-inhibitor (PI) bacteria had limited effects (Figures 1A, B). These findings suggest that in complex microbial environments where PH bacteria coexist with pathogens, suppressing PH may be more efficient than directly targeting the pathogen.

In agriculture, *Ralstonia solanacearum*, the causative agent of bacterial wilt, has a severe impact on crops in the Solanaceae family. A recent study identified two PH bacteria, *Phyllobacterium ifriquityense* and *Microbacterium paraoxydans*, from the tomato rhizosphere microbiome that exacerbate the disease (Li et al., 2022). Beyond these two helpers, more than half of the isolated rhizobacteria facilitated pathogen growth, indicating a widespread presence of PH bacteria. Additionally, this study identified numerous PI, as well as IPH, bacteria. Surprisingly, managing pathogens indirectly by targeting PH reduced disease symptoms more effectively than direct pathogen control (Figure 1C). These findings underscore the potential of using IPH for indirect pathogen control during biological management of phytopathogens.

Another study discovered two noteworthy PI rhizobacteria, *Niallia* sp. RD1 and *Pseudomonas putida* H3, which mitigate bacterial wilt in tomatoes (Lee et al., 2024). RD1, a beneficiary, cannot grow independently but thrives when co-cultured with H3, a helper, that supplies succinate. Interestingly, growth of H3 also benefits from RD1, highlighting a mutualistic syntrophic interaction (Figure 1C). Their collaboration reduced bacterial wilt more effectively than individual treatments. This study examines the potential synergistic effects of two PI bacteria in agriculture and suggests that identifying new helpers of PI could be important for effective disease control.

Nevertheless, implementing indirect strategies in practice faces several challenges. Indirect interventions may be slower to manifest and require more complex implementation compared to direct methods. Environmental variables such as soil composition can influence the effectiveness of these interactions, affecting reproducibility across different settings. Moreover, scaling up such strategies for large-scale agricultural or clinical use requires careful consideration of economic feasibility, regulatory frameworks, and potential unintended consequences on the microbiome and ecosystem. Addressing these challenges necessitates interdisciplinary collaboration and comprehensive research to develop robust, adaptable solutions.

## Levels of indirect inhibition

Here, we suggest hierarchical levels of indirect interactions. The interpretation of the examples of indirect interaction-based inhibition of pathogen infection leads us to explore the different levels of these interactions. As shown in Figure 1D, the first level involves inhibiting bacteria that target the growth of PH bacteria. Theoretically, under *in situ* conditions, additional indirect interactions, referred to as Level 2, occur and connect with Level 1. In Level 3, linear and multiple indirect interactions may establish a two-dimensional (single-layer) network of indirect interactions. The association of this single layer leads to formation of multiple orbits, resulting in multiple layers of indirect interaction.

Understanding these levels is crucial for developing effective strategies for pathogen control. However, as the complexity of the interaction network increases, the difficulty in predicting outcomes and implementing practical interventions correspondingly escalates. Methodological biases in network analyses can misrepresent the importance of certain species, and the dynamic nature of microbial communities influenced by environmental variables adds further uncertainty. To overcome these challenges, integrating multi-omics data with experimental validation is essential. Advanced computational models that account for environmental factors and microbial adaptability can improve prediction accuracy. Moreover, field trials under diverse conditions are necessary to assess the real-world applicability and robustness of these indirect interaction strategies.

## Open questions and potential solution

Traditionally, experimental limitations have forced microbiologists to focus on direct inter- and intra-species interactions. Reductionism has often been regarded as the logical approach to understanding these interactions; however, studies on gut and soil microbiomes show that interactions are rarely simple one-to-one events. In bacterial communities, it is uncommon for all bacteria to engage with each other directly. Instead, interactions are often multi-layered, involving secondary and tertiary connections (Figure 1D). This is analogous to research in human social networks, which has identified hubs and nodes with varying degrees of connectivity. These concepts have been applied to microbial interactions through specific algorithms and computational tools; however, biologically validating these second interactions results in several open questions: (1) What are the key determinants of interaction? (2) How can target microbes and their interacting partners be isolated and identified? (3) Are there established *in vitro* and *in situ* systems for validation? (4) How can clinical or field experiments be conducted? Below, we propose potential solutions to these questions:

1. Key determinant(s) of interactions: identifying the key factors that drive indirect interactions is essential. Potential candidates include nutrients that serve as common goods, or rare food sources crucial for microbial growth. Integrating metabolomics and metatranscriptomics analyses to study interactions can help identify the key determinants of target microbes.
2. Identification and isolation of interacting microbes: to evaluate indirect interactions, it is essential to isolate the relevant microbes after identifying the key determinants; however, isolating the appropriate species can be challenging. Developing novel culture systems, including new media, identifying previously unknown growth factors, and refining anaerobic cultivation techniques, is necessary to align cultured isolates with metagenomic data. Advancements in single-cell genomics and microfluidic technologies can facilitate the cultivation and study of previously uncultivable microbes. Utilizing co-culture systems that mimic natural environments can also aid in isolating interacting partners.
3. *In vitro* and *in vivo* validation of isolated microbes: the fitness and growth of isolated microbes should be assessed in relation to their interaction partners. Both defined and complex media can be used for *in vitro* assays, and the inhibition of target microbe growth in tritrophic interactions must be demonstrated. *In vivo* validation can be conducted using model animals or organoid-based systems. Developing standardized microcosm and mesocosm models that replicate natural ecosystems can enhance the relevance of laboratory findings. Moreover, advanced imaging techniques and molecular probes can help monitor interactions in real-time within these systems.
4. Clinical and field application: promising microbes identified through *in vitro* and *in vivo* validation can be advanced to clinical trials or field applications; however, systems for evaluating clinical trials based on indirect interactions are not yet well-established, and further research is needed to determine the effectiveness of these approaches in real-world settings. This remains a significant challenge. Collaborative efforts with stakeholders, including farmers, clinicians, and policymakers, are essential. Establishing pilot programs and demonstration sites can provide valuable data on effectiveness and feasibility. In addition, regulatory frameworks need to be developed to ensure safety and compliance with environmental and health standards.

## Concluding remarks

Microbial communities operate through complex networks of both direct and indirect interactions. While direct antagonism has long served as the foundation for antibiotic discovery and microbial disease control, it does not fully represent the ecological complexity of natural environments. Shifting the focus to indirect modulation, such as targeting nonpathogenic microbial partners that influence pathogen behavior, opens new directions for sustainable and effective control strategies in both medical and agricultural settings. This perspective promotes a more integrated understanding of microbial ecosystems, where indirect interactions can act as major regulators of pathogenic processes.

However, this approach presents important limitations. Analytical bias in microbial network reconstruction, the influence of fluctuating environmental conditions, and the absence of experimental validation often constrain the practical application of indirect strategies. In addition, many microbes involved in indirect inhibition are low in abundance and difficult to isolate or culture, making mechanistic investigation and real-world deployment more difficult. The limitations emphasize the need for improved methodological frameworks that can incorporate microbial complexity, environmental context, and host specificity.

Future studies should focus on validating indirect interactions in controlled settings using microbial consortia, time resolved monitoring, and advanced imaging techniques. The integration of metagenomics, metabolomics, and transcriptomics will help clarify the functional mechanisms of indirect inhibition. Clinical and field level applications must also consider ecological safety, regulatory challenges, and cost effectiveness.

Ultimately, focusing on indirect ecological interactions represents a meaningful shift from reductionist thinking to systems level understanding. This transition requires collaboration across microbiology, ecology, computational biology, and field practice. With support from deep learning and artificial intelligence, researchers can identify and target specific microbial interactions, enabling more precise and context responsive disease control strategies. By addressing current limitations and advancing this framework, indirect interaction-based approaches have the potential to support resilient and long term solutions for managing infectious diseases in complex microbial environments.

## Data Availability

The original contributions presented in the study are included in the article/supplementary material, further inquiries can be directed to the corresponding author.
